# VEGF signaling in acute leukemia: mitochondrial connections

**DOI:** 10.18632/oncoscience.400

**Published:** 2018-04-29

**Authors:** Sandrina Nóbrega-Pereira, Sérgio Dias

**Affiliations:** Instituto de Medicina Molecular João Lobo Antunes, Faculdade de Medicina, Universidade de Lisboa, Lisboa, Portugal

**Keywords:** leukemia, VEGFR2, chemotherapy-resistance, mi-tochondria, PGC-1α, metabolism

The vascular endothelial growth factor (VEGF) signaling pathway regulates acute myeloid leukemia (AML) cell proliferation, survival and chemotherapy resistance. We have shown that VEGF/VEGFR2 signaling regulates leukemia cell survival by autocrine internal and external loops, with induction of pro-survival pathways and inhibition of apoptosis downstream of VEGFR2 internal signaling [1, 2]. Moreover, VEGF signaling can mediate paracrine vascular endothelial cell-controlled angiogenesis in AML, promoting vessel formation and leukemic blasts maintenance. As a consequence, aberrant VEGF signaling operates in the bone marrow of AML patients and is related to a poor prognosis, constituting an attractive target for therapeutic intervention in AML. Several therapeutic strategies (including monoclonal antibodies against VEGF-A and VEGFR-2 tyrosine kinase inhibitors) have been tested in clinical trials for refractory and relapsed AML with slight beneficial effects in combination with chemotherapy. The implication of VEGF/VEGFR2 signaling in multiple processes (including hematopoiesis and vascular growth in physiological and pathological situations) demands a deeper understanding of the VEGF/VEGFR2 downstream effectors operating specifically in leukemia cells as viable targets for combinatorial therapy particularly for relapsed AML patients.

Our recent study [3] reveals a previously undisclosed VEGFR-2 target that regulates the response of acute leukemia to chemotherapy. Using a novel mouse xenograft model of human AML that enabled chemotherapy-induced regressions of established disease followed by lethal regrowth, we realized that human AML cells from terminally ill mice treated with chemotherapy (chemoAML) exhibited enhanced VEGF/ VEGFR2 signaling and several metabolic alterations, including fewer mitochondria and reduced expression of mitochondrial biogenesis factors. Profound changes in cellular metabolism have been shown to take place during acute leukemia pathogenesis, progression and disease recurrence whereas the signaling pathways that orchestrate those adaptations are less characterized. A putative role for VEGF/VEGFR2 signaling in controlling the metabolic plasticity of leukemia cells had not been established. Importantly, autocrine VEGF signaling has been shown to regulate anabolic metabolism and survival of endothelial cells [4].

With all this in mind, we reasoned that VEGFR2 signaling could counteract chemotherapy-response by regulating mitochondrial metabolism. Accordingly, we show that VEGFR2 inhibition restored mitochondrial biogenesis and function and, importantly, increased the cell-death response of chemoAML cells to chemotherapy, revealing that VEGFR2 signaling modulates the response of AML cells to chemotherapy by controlling mitochondrial mass and biogenesis. The development of resistance to anti-angiogenic drugs, targeting VEGFR among other receptors, is accompanied by an increase reliance on mitochondrial respiration that drives solid tumor survival [5]. Collectively, these findings suggest that repression of mitochondrial metabolism may be a general outcome of VEGFR2 signaling in cancer cells, resulting in pro- or anti-tumorigenic outcomes depending on the metabolic (and other) requirements of the tumor.

**Figure 1:**
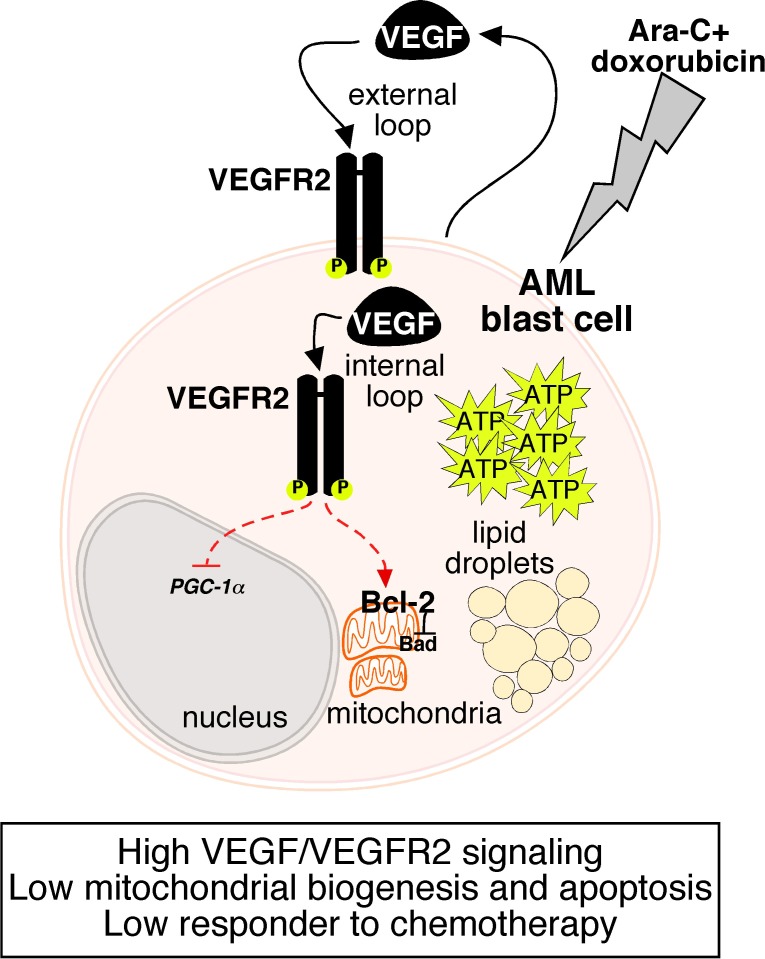
VEGFR2–mediated reprogramming of mitochondrial metabolism regulates the sensitivity of AML cells to chemotherapy Enhanced VEGF/VEGFR2 autocrine internal signaling counteracts the response of AML cells to chemotherapy (combined treatment of Ara-C and doxorubicin) by maintaining a low mitochondrial mass and apoptotic function through repression of PGC-1α and a high Bcl-2:Bad ratio, resulting in overall improved leukemia cell viability. Chemotherapy-exposed AML cells present additional metabolic traits such as increased lipid droplets and ATP levels.

There is an increasing appreciation of the importance of targeting mitochondria in acute leukemia. Enhanced mitochondrial metabolism is believed to represent a liability due to the role of mitochondria in apoptosis and generation of reactive oxygen species. On the other hand, increased reliance on lipid metabolism and mitochondrial respiration has been described in leukemia relapses, highlighting the CD36-FAO-OXPHOS axis as a plausible target to eradicate therapy-resistant leukemia [6, 7]. In our study, we show that chemotherapy (combined treatment of arabinofuranosyl cytidine [Ara-C] and doxorubicin)- exposed AML cells present increased lipid content, higher lactate, pyruvate and ATP levels, a metabolic signature compatible with enhanced glycolysis and metabolic plasticity. Several lines of evidence suggest a link between glycolytic activity and therapy-resistance in AML; with inhibition of glycolysis increasing Ara-C sensitivity in AML primary blasts [8]. Moreover, we show that mitochondrial metabolism is required for the sensitization to chemotherapy provided by VEGFR2 inhibition in the absence of overt changes in other metabolic features but relying on a low Bcl- 2:Bad protein ratio. These findings support the notion that VEGFR2 signaling decreases the sensitivity of AML cells to chemotherapy by reprogramming mitochondrial bioenergetic and apoptotic functions, resulting in overall improved tumor cell viability (Figure 1).

Mechanistically, we show that VEGFR2 inhibition elicited a transcriptional response in chemotherapy- exposed AML cells, resulting in increased expression of *PGC-1α*, *PGC-1β*, *ERRα*, and *NRF1*, known regulators of oxidative metabolism and mitochondrial biogenesis. Importantly, we show that low levels of *PGC-1α* expression are associated with worst overall AML patient survival, and in chemotherapy-exposed AML cells, PGC- 1α depletion abolished the capacity of VEGFR2 inhibition to re-sensitize to chemotherapy treatment, highlighting PGC-1α as a molecular suppressive axis for VEGFR2- induced viability in therapy-resistant AML cells (Figure 1).

A current challenge of designing new combinatorial therapies for AML is the diversity of genetic abnormalities found at diagnosis, making the identification of a common target among AML subgroups an urgent need. Our results lend support for the development of PGC-1α activators as a suitable alternative for new combinatorial therapies in treating relapsed AML. As opposed to blocking VEGFR2 *per se*, this strategy may confer another layer of specificity by directly suppressing a leukemic-specific downstream target without interfering with VEGFR2 signaling in normal cells or AML-associated angiogenesis. It will be important to determine if PGC-1α activation is a viable therapeutic modality to increase the response to chemotherapy in multiple therapy-resistant AML subgroups or only for VEGFR2-dependent patients subsets. Further studies are warranted but a new avenue for combinatorial strategies to treat therapy-resistant leukemia is now open.
